# Supplementary figures and images for: Covariation Between Microbiome Composition and Host Transcriptome in the Gut of Wild Drosophila melanogaster: A Re‐Analysis
Source: Ecol Evol. 2025 Jan 12;15(1):e70853. doi: 10.1002/ece3.70853 (PMC11725384; doi:10.1002/ece3.70853)

## Gut Microbiome

## Host Gut Transcriptome

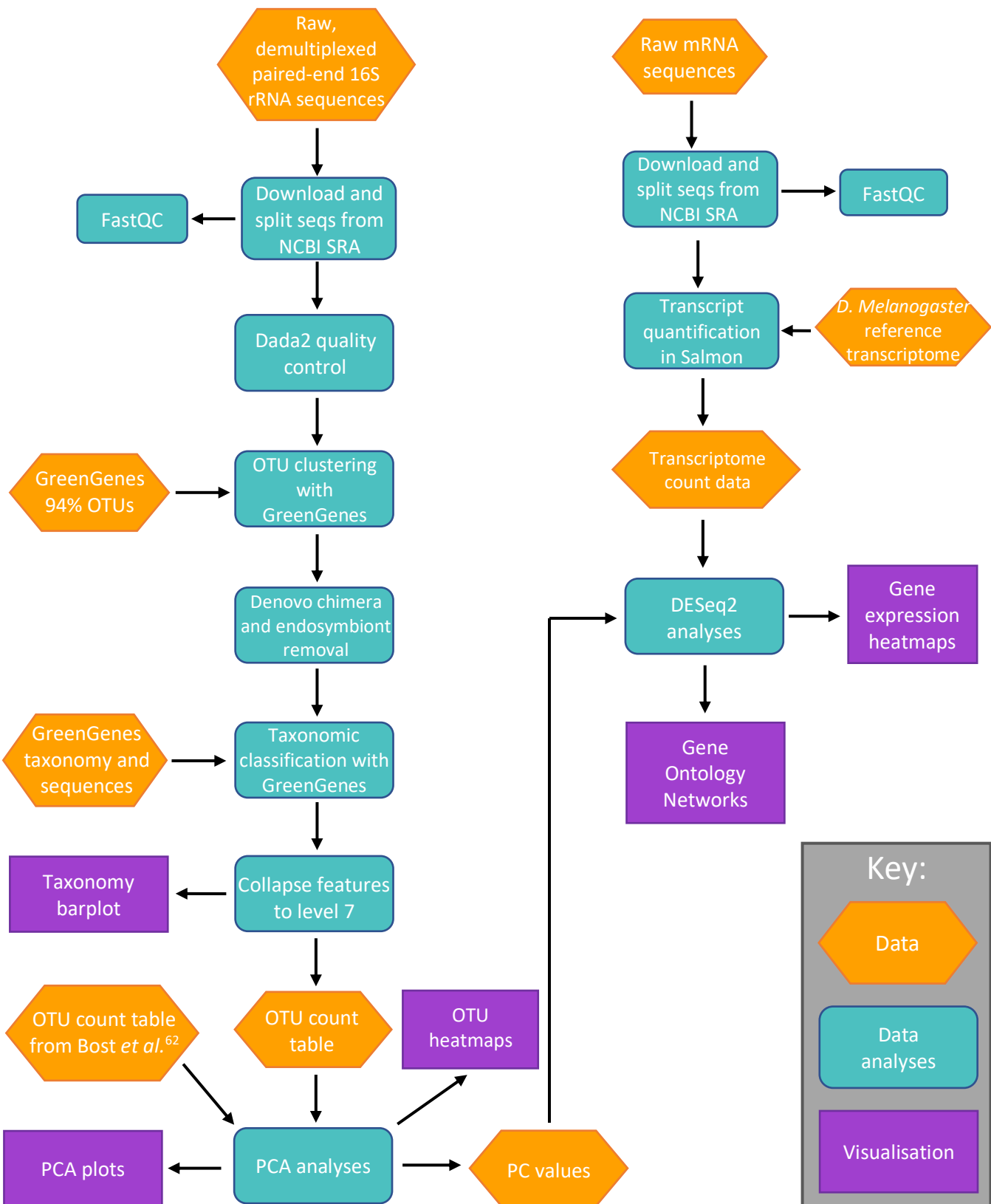

Supplement: Supplementary file 1 — Figure S1. Detailed view of analysis. Figure integrates steps taken between (Bost, Franzenburg, et al. 2018) and the present study. [file ECE3-15-e70853-s005.pdf]

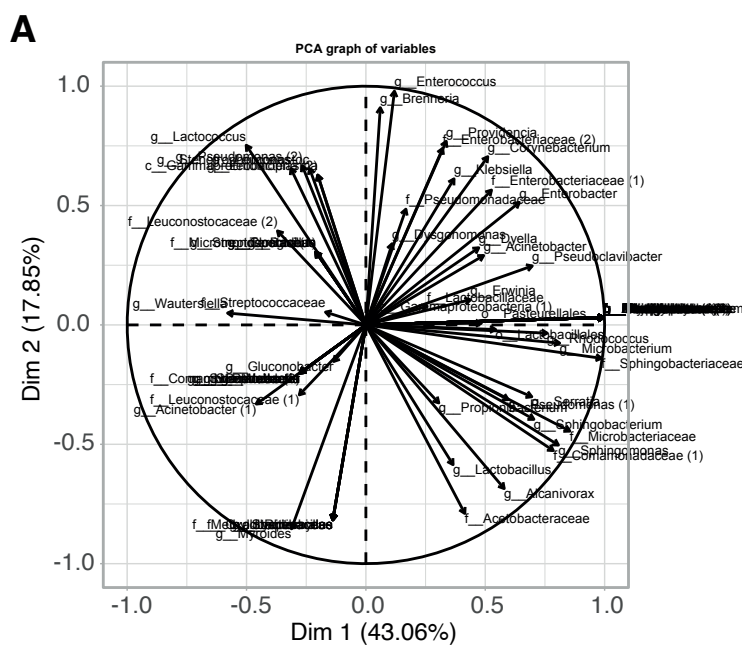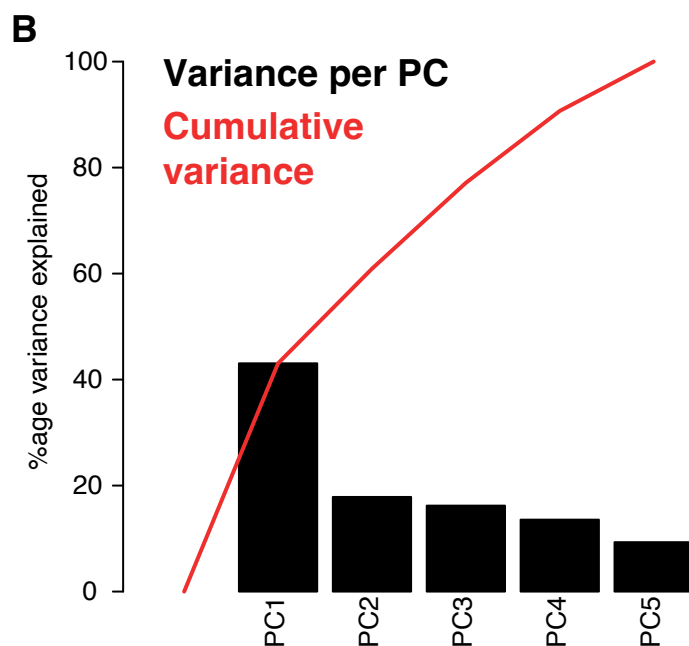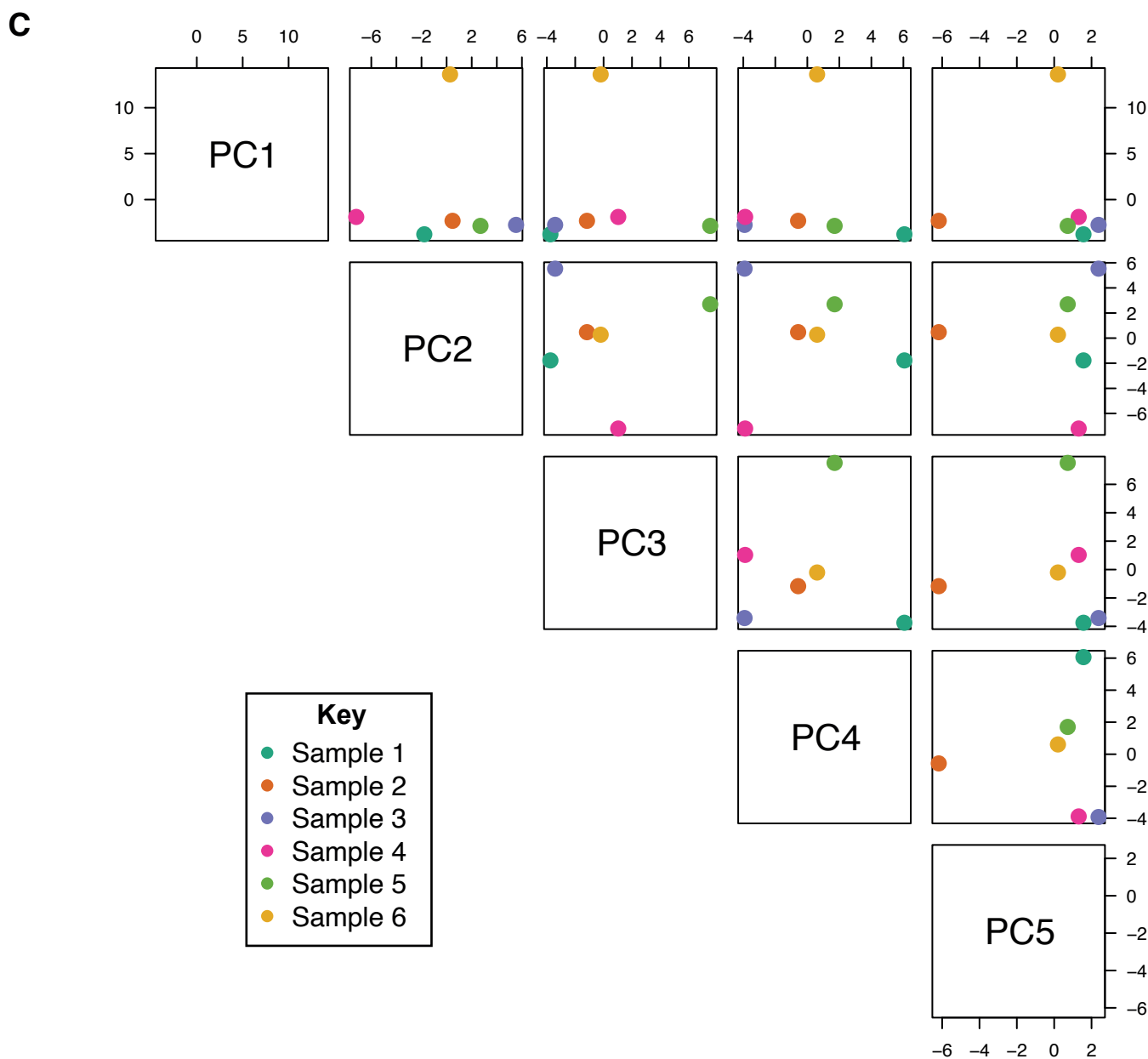

Supplement: Supplementary file 2 — Figure S2. Principal components analysis of ASVs reveals orthogonal axes of variation in wild fly microbiota. (A) Biplot showing rotation of ASVs on first two principal components (PCs). (B) Variance explained by each PC. (C) Lack of correlation between PCs indicates orthogonal axes of microbiome variation. Pairs plot showing positions of each sample on pairs of PCs, for all pairwise combinations. Panels show positions on PCs shown to left (Y axis) and below (X axis), for example, top‐right panel shows positions on PC1 and PC5. [file ECE3-15-e70853-s001.pdf]

Figure S3

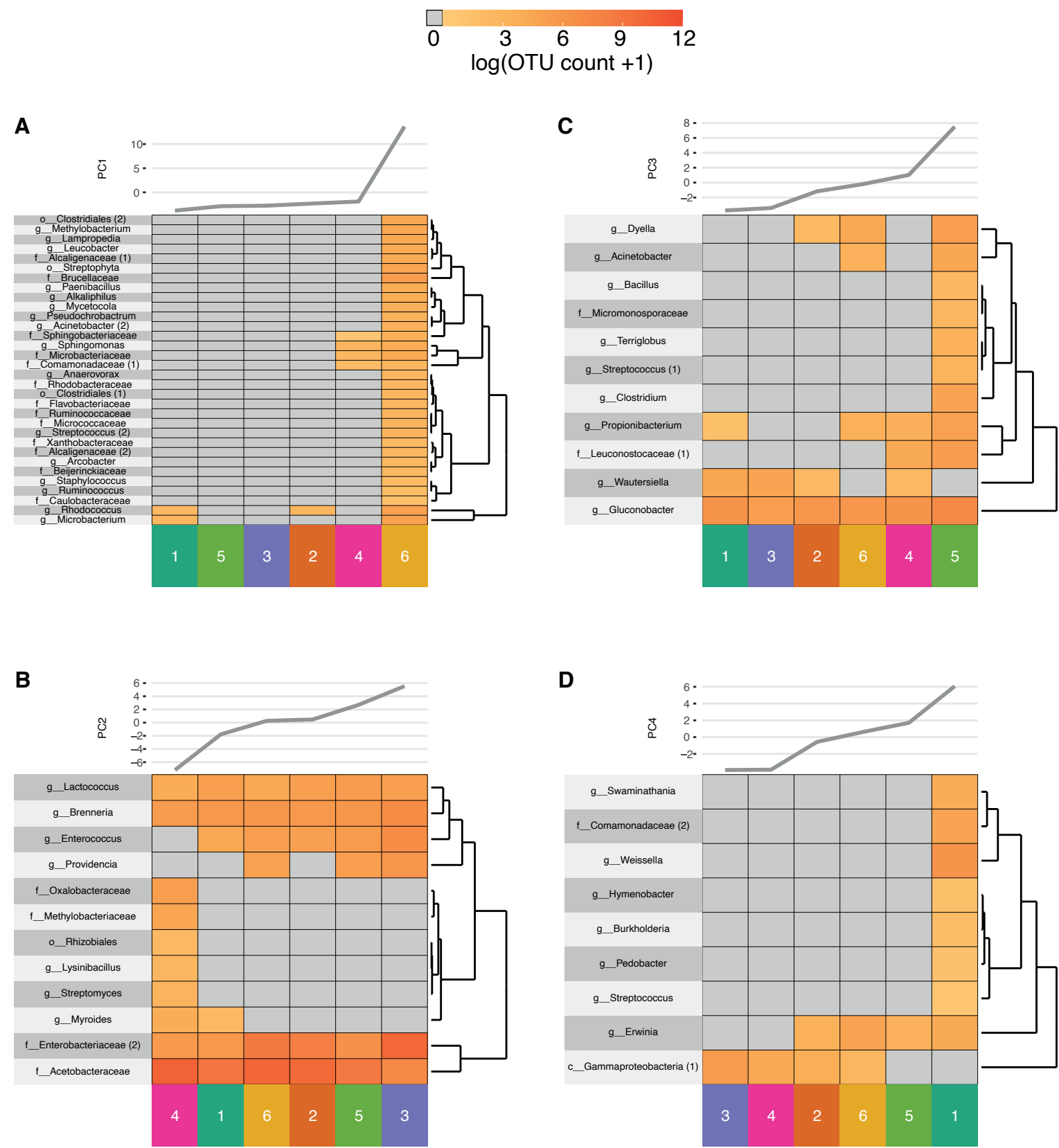

Supplement: Supplementary file 3 — Figure S3. Mapping microbiome PC values to axes of variation in ASV occurrence and abundance. Major axes of variation in ASV counts were identified by principal components analysis. Resulting PCs were mined for correlations with starting ASV table, identifying ASVs putatively underlying each PC. Panels A–D show heatmap analysis for ASVs associated to PCs 1–4, respectively. Heatmaps show values for each PC (line plots at top) and ASV counts (natural log). Sample identities are given at the bottom. Samples are ordered by microbiota PC values (at top). Rows are ordered by hierarchical clustering (dendrograms to right of figures). [file ECE3-15-e70853-s009.pdf]

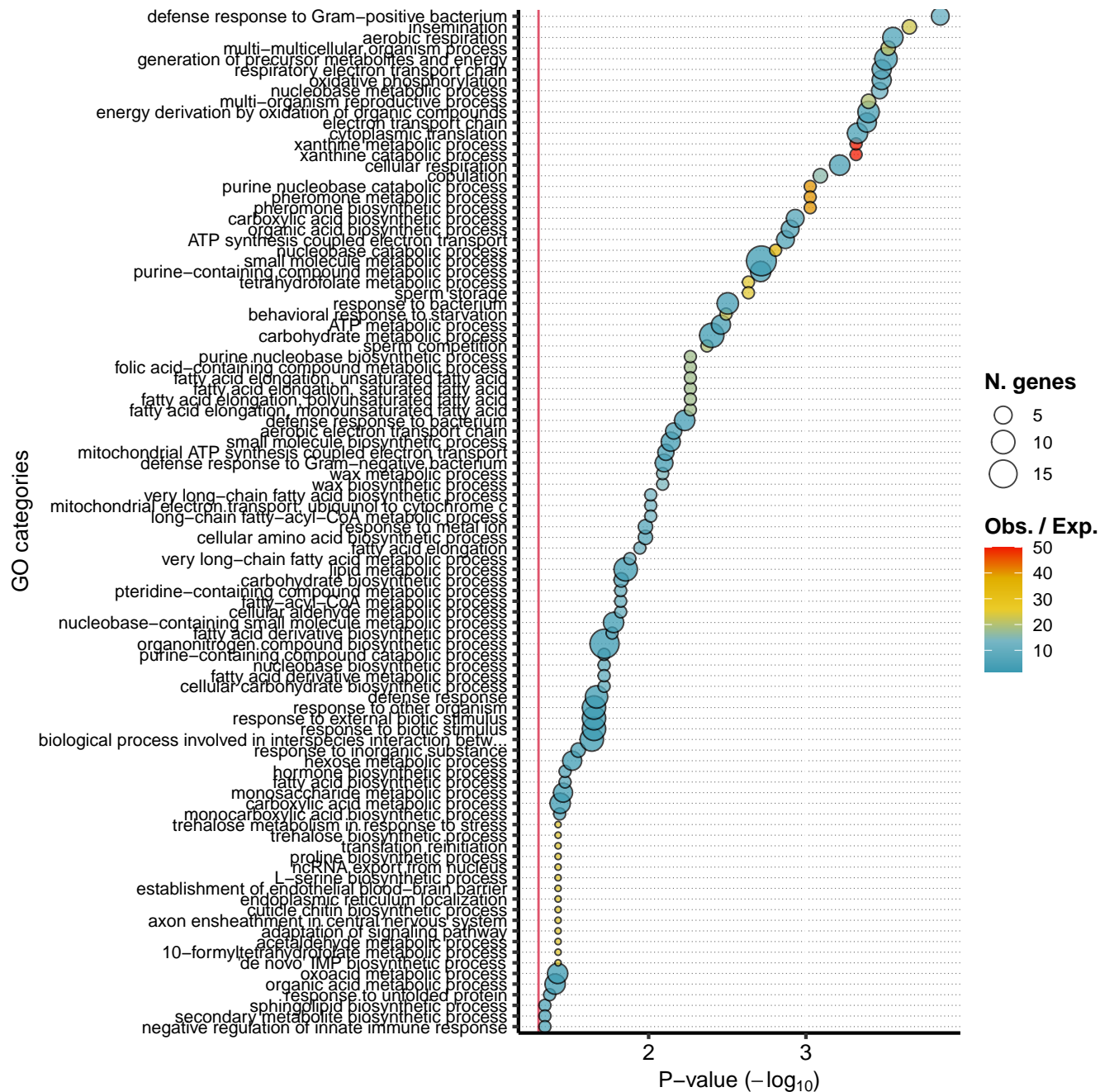

Supplement: Supplementary file 4 — Figure S4. Biological process gene ontology (GO) terms in the host gut transcriptome associated with taxonomic variation in the wild fly microbiota. GO enrichment was calculated for the full set of all genes associated to microbiome PCs 1–4. Bubble plots show enrichment of GO terms per PC and per ontology. X axis shows p values (Fisher’s test) for enrichment. GO terms are given on Y axis, ordered by X axis values. Bubble size shows number of genes in the set associated with the GO term and colour shows ratio of n. observed/expected genes. [file ECE3-15-e70853-s008.pdf]

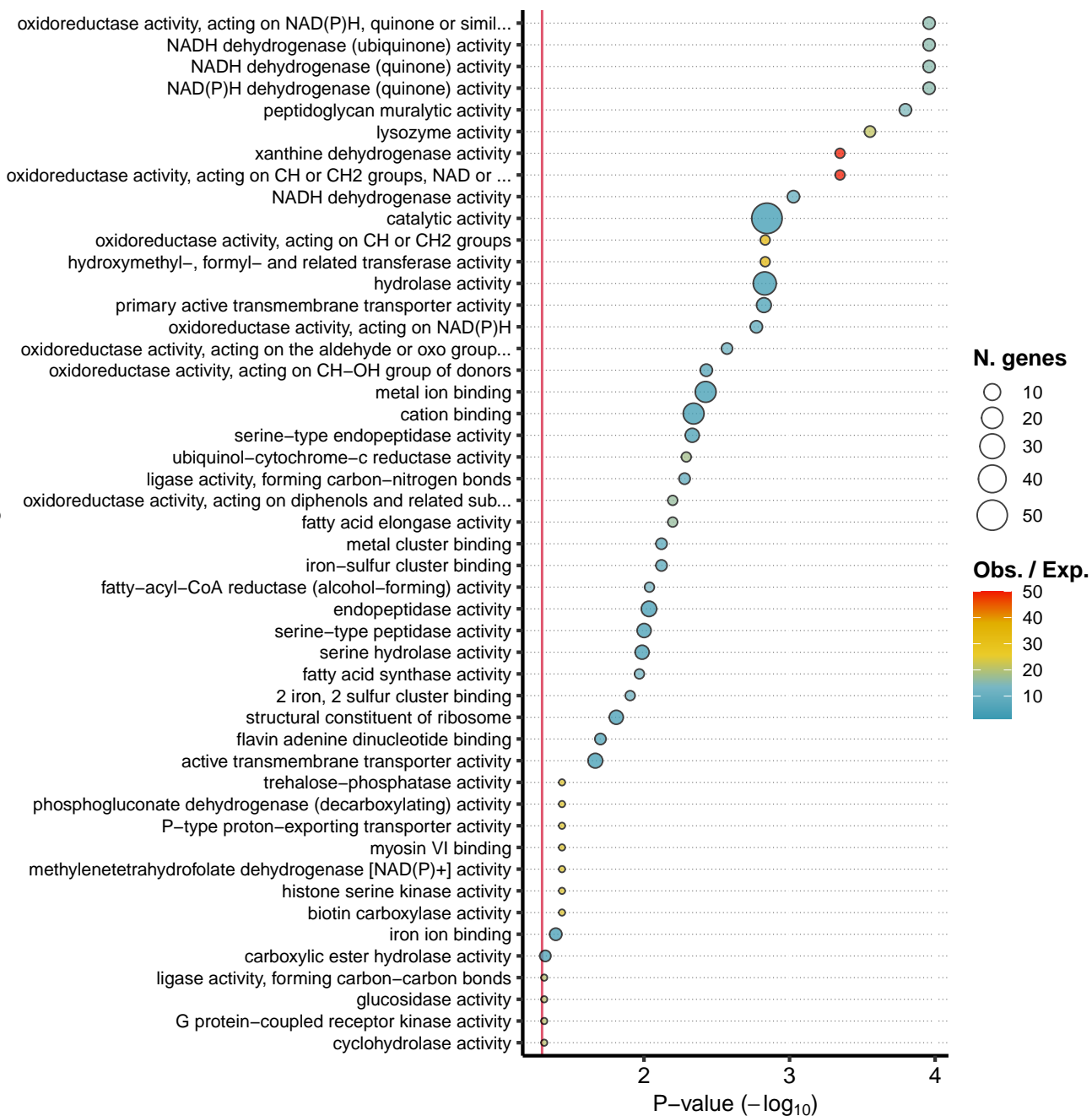

Supplement: Supplementary file 5 — Figure S5. Molecular function gene ontology (GO) terms in the host gut transcriptome associated with taxonomic variation in the wild fly microbiota. GO enrichment was calculated for the full set of all genes associated to microbiome PCs 1–4. Bubble plots show enrichment of GO terms per PC and per ontology. X axis shows p values (Fisher’s test) for enrichment. GO terms are given on Y axis, ordered by X axis values. Bubble size shows number of genes in the set associated with the GO term and colour shows ratio of n. observed/expected genes. [file ECE3-15-e70853-s003.pdf]

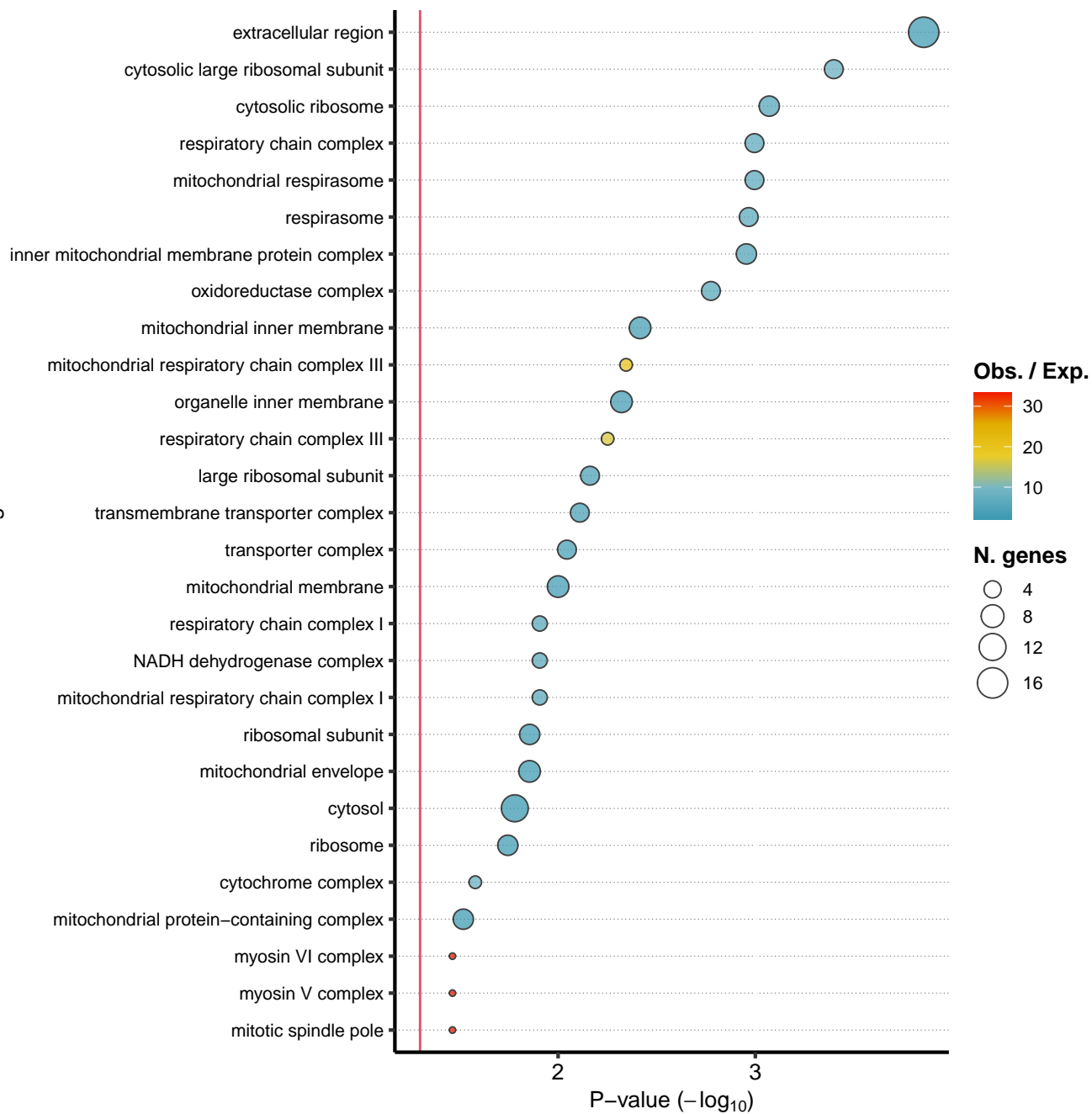

Supplement: Supplementary file 6 — Figure S6. Cellular component gene ontology (GO) terms in the host gut transcriptome associated with taxonomic variation in the wild fly microbiota. GO enrichment was calculated for the full set of all genes associated to microbiome PCs 1–4. Bubble plots show enrichment of GO terms per PC and per ontology. X axis shows p values (Fisher’s test) for enrichment. GO terms are given on Y axis, ordered by X axis values. Bubble size shows number of genes in the set associated with the GO term and colour shows ratio of n. observed/expected genes. [file ECE3-15-e70853-s004.pdf]

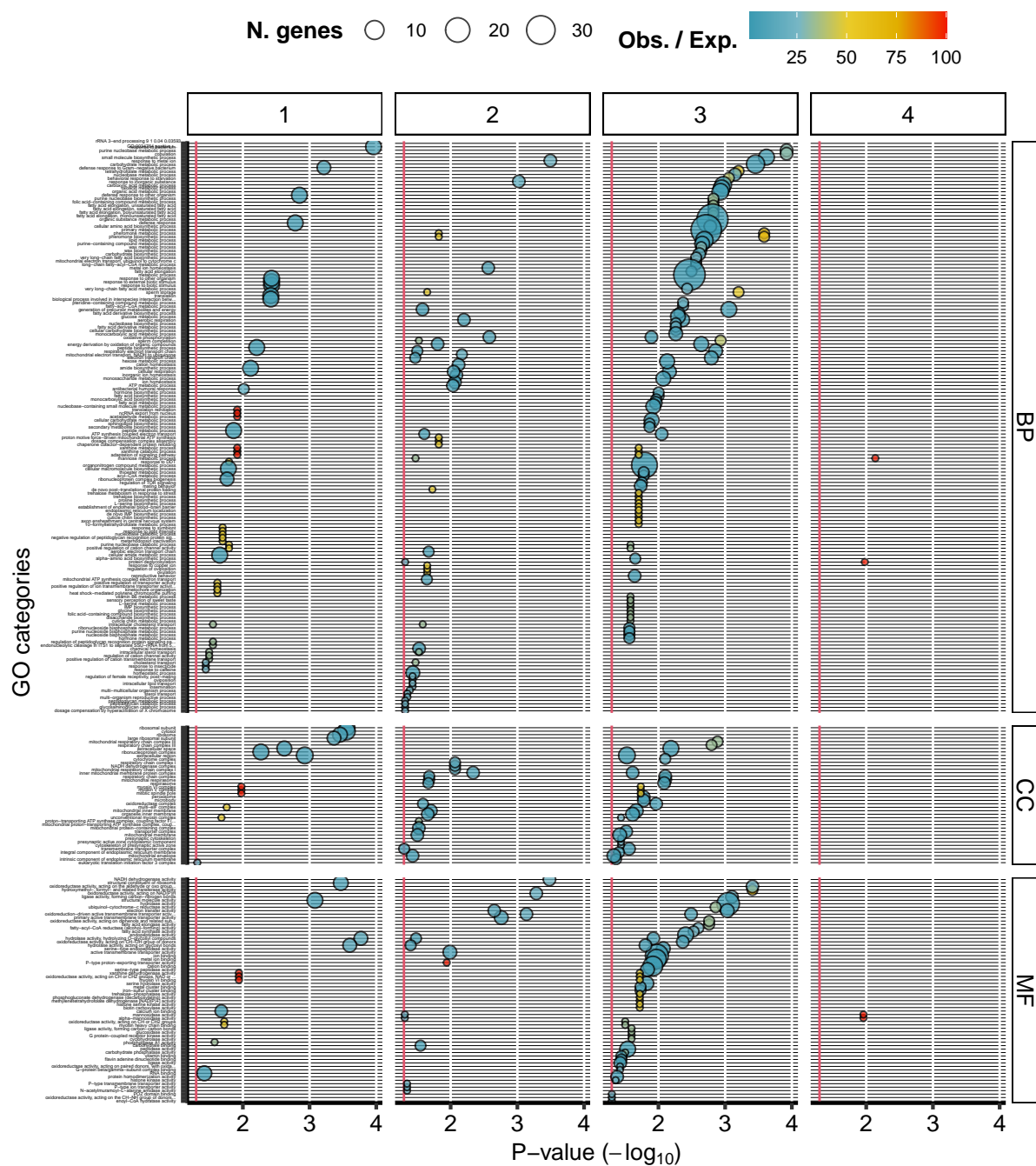

Supplement: Supplementary file 7 — Figure S7. Distinct host functions associated to orthogonal axes of microbiota variation. Gene ontology (GO) enrichment was calculated for gene sets associated to microbiome PCs 1–4, for biological process (BP), cellular component (CC) and molecular function (MF) ontologies. Panel shows enrichment per gene set (columns) and ontology (rows). Bubble plots show enrichment of GO terms per PC and per ontology. X axis shows p values (Fisher’s test, −log10 scale) for enrichment. GO terms are given on Y axis, ordered by average of X axis values across all gene sets. Bubble size shows number of genes in the set associated with the GO term and colour shows ratio of n. observed/expected genes. All GO terms per each gene set are shown. See Tables S7–S10 for detail of GO terms, and Figure 5 for an equivalent plot of the top 5 GO terms per gene set. [file ECE3-15-e70853-s002.pdf]
